# Metastatic Uterine Leiomyosarcoma in the Upper Buccal Gingiva Misdiagnosed as an Epulis

**DOI:** 10.1155/2014/402342

**Published:** 2014-10-15

**Authors:** Andrea Cassoni, Valentina Terenzi, Davina Bartoli, Oriana Rajabtork Zadeh, Andrea Battisti, Mario Pagnoni, Davide Conte, Alessandro Lembo, Sandro Bosco, Francesco Alesini, Valentino Valentini

**Affiliations:** ^1^Maxillofacial Surgery, Odontostomatological Science and Maxillofacial Surgery Department, “Sapienza” University of Rome, Viale del Policlinico 155, 00100 Rome, Italy; ^2^Medical Oncology, Clinica Marco Polo, Viale Marco Polo 41, 00100 Rome, Italy; ^3^Molecular Medicine Department, “Sapienza” University of Rome, Viale del Policlinico 155, 00100 Rome, Italy

## Abstract

Uterine leiomyosarcoma (LMS) is a rare tumor constituting 1% of all uterine malignancies. This sarcoma demonstrates an aggressive growth pattern with an high rate of recurrence with hematologic dissemination; the most common sites are lung, liver, and peritoneal cavity, head and neck district being rarely interested. Only other four cases of metastasis in the oral cavity have been previously described. The treatment of choice is surgery and the use of adjuvant chemotherapy and radiation has limited impact on clinical outcome. In case of metastases, surgical excision can be performed considering extent of disease, number and type of distant lesions, disease free interval from the initial diagnosis to the time of metastases, and expected life span. We illustrate a case of uterine LMS metastasis in the upper buccal gingiva that occurred during chemotherapy in a 63-year-old woman that underwent a total abdominal hysterectomy with bilateral salpingo-oophorectomy for a diagnosis of LMS staged as pT2bN0 and that developed lung metastases eight months after primary treatment. Surgical excision of the oral mass (previously misdiagnosed as epulis at a dental center) and contemporary reconstruction with pedicled temporalis muscle flap was performed in order to improve quality of life. Even if resection was achieved in free margins, “local” relapse was observed 5 months after surgery.

## 1. Introduction

Uterine leiomyosarcomas (LMSs) constitute 1% of all uterine malignancies and approximately 25% of uterine sarcomas, that are rare tumors that account for only 3% to 7% of all uterine cancers [1, 2]. They demonstrate an aggressive growth pattern with a high rate of recurrence with hematologic dissemination; the most common sites are lung, liver, and peritoneal cavity [3]. Head and neck district is rarely interested, and only other four cases of metastasis in the oral cavity have been previously described [4]. In particular, we illustrate the second case of metastasis in the upper buccal gingiva in a 63-year-old woman in which lung metastasis was also present.

## 2. Case Report

**Figure 1:**
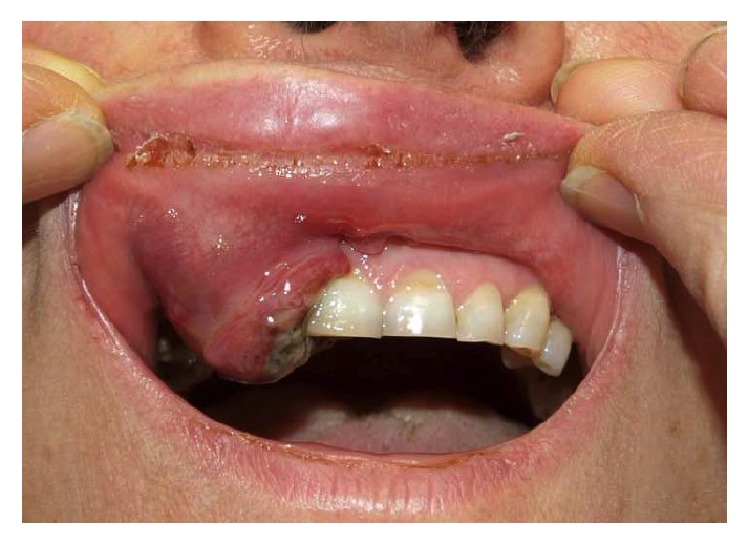
Preoperative intraoral view of the lesion.

**Figure 2:**
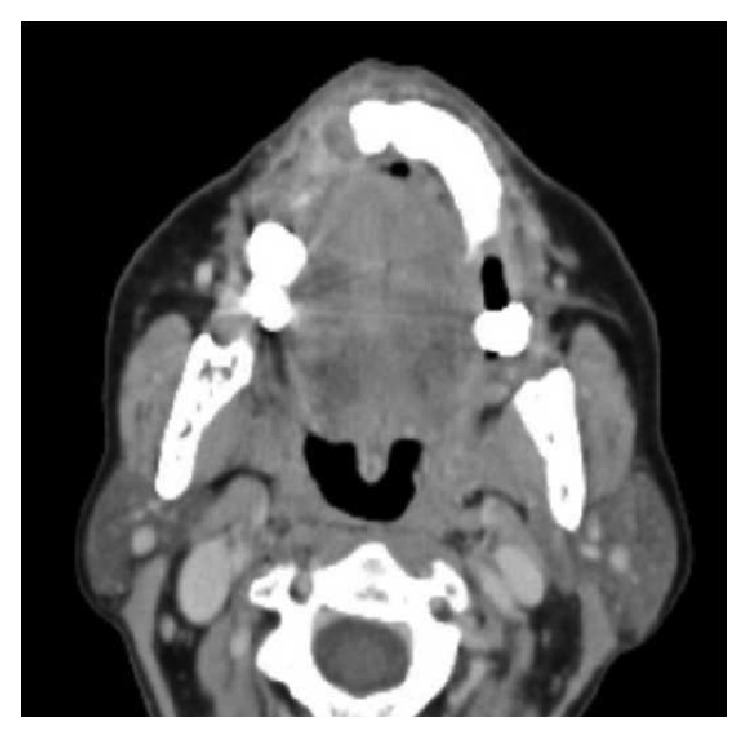
Preoperative axial CT scans with contrast showing left upper alveolar crest lesion.

**Figure 3:**
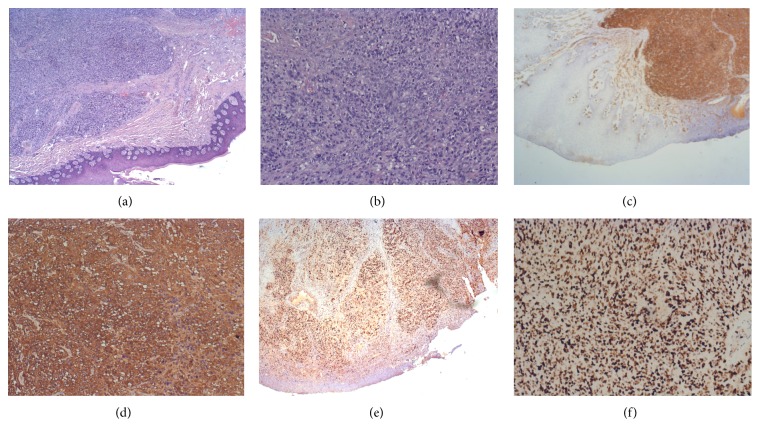
(a) Hematoxylin eosin stain of the specimen (2x HPF). (b) Hematoxylin eosin stain of the specimen (10x HPF). (c) Immunohistochemistry showing positivity to a-smooth muscle actin (a-SMA) (2x HPF). (d) Immunohistochemistry showing positivity to a-smooth muscle actin (a-SMA) (10x HPF). ((e) and (f)) Immunohistochemistry revealing a mitotic index (Ki-67) of 90% positivity (2x HPF and 10x HPF).

A 63-year-old woman with a history of advanced uterine LMS presented to our center with a painful, ulcerated swelling on the right upper buccal gingiva (Figure 1). She underwent a total abdominal hysterectomy with bilateral salpingo-oophorectomy for a diagnosis of LMS staged as pT2bN0 fourteen months previously. Eight months after primary treatment she developed two bilateral lung masses (SUV max: 7.8 at (18)F-fluoride PET/CT); histological examination of the 11 mm lung mass in the right chest revealed a metastasis of LMS. An adjunctive lung mass was present in the left chest. Chemotherapy was administered, initially with isofosfamide-epidoxorubicin, with scarce tolerance (grade 3-4 neuthropenia) so that it was decided to administer taxotere. During this treatment she developed a rapidly growing mass in the right upper buccal gingiva. She was referred to her dentistry that performed an excisional biopsy under local anesthesia suspecting a “haemorragic epulis.” Histological examination was compatible with metastasis of leiomyosarcoma, incompletely excised. A CT scan performed about 1 month after resection revealed an osteolytic lesion of 30 × 35 mm of the upper maxilla (Figure 2); MRI was not performed because the patient was claustrophobic. Because of the rapid growth observed just in 40 days, it was decided according to oncologist to perform a partial maxillectomy and contemporary reconstruction with temporalis muscle pedicled flap even if a lung metastasis was present. Histological examination confirmed the presence of a 6 cm spindle cells LMS, entirely excised. Immunohistochemical analysis against monoclonal antibodies for a-smooth muscle actin (a-SMA), desmin, HHF-35, caldesmon, and vimentin was positive; mitotic index (Ki67) was of 90% (Figure 3). Considering the radical excision, no “adjuvant” radiotherapy on the oral cavity was administered. In order to control lung metastasis and considering the aggressiveness of the disease, it was decided to perform two other cycles of chemotherapy with ifofosfamide-epidoxorubicin. A partial regression of the lung metastasis was obtained, but again it was impossible to continue treatment because of high toxicity. A PET-CT performed 3 months after surgery revealed a right femur metastasis, so that radiotherapy was performed. Two months later, after an episode of epistaxis, head and neck CT scan with contrast revealed a relapse in the masticatory space and rinopharynx: the patient was referred to palliative radiotherapy.

## 3. Discussion

LMSs usually arise in anatomical sites with abundant smooth muscle, such as gastrointestinal tract, retroperitoneum, and uterus; nevertheless, uterine LMSs constitute only 1% of all uterine malignancies [1, 2, 4]. Despite variable results, patients with uterine LMS have an overall poor prognosis with a long-term 5-year survival and risk of recurrence ranging from 25% to 75%, even if these tumors are usually confined to the uterus at the time of diagnosis [5, 6]. The majority of patients who recur do so within 2 years of initial diagnosis, and up to 90% of patients who fail show distant metastases either alone or concurrent with pelvic recurrence. In our case, the tumor was confined to the uterus at the time of diagnosis, and distant metastases to the lung were detected eight months after primary treatment. The most common sites of recurrence are peritoneal cavity (30–50% of cases), lung (30–40%), and liver (about 10%); only rarely do they involve the brain and skull (<1%) owing to pulmonary arterial circulation [7]. Metastatic uterine LMS to the oral cavity are extremely rare, and only four other cases have been previously described [4, 8]. Also considering LMSs originated from other primary sites, only other three cases with metastasis to oral cavity can be found [8, 9]. The rarity of buccal mucosa involvement can be due to the fact that in the oral cavity smooth muscle is very scarce, being present in the blood vessel walls and the circumvallate papillae of the tongue. Kim et al. hypothesize that “the metastatic mass must be related to the blood vessels in the buccal cheek, and the maxillary gingivae with haematogenous spread from the uterus via lung to the oral cavity” [4]. In our case oral metastasis to the upper gingival developed after lung metastases. They also found that metastatic tissue was different from tissue from primary site, being more vascularized and composed by cells more spindle-shaped moderately positive to the a-SMA antibody [4]. In our case it was impossible to compare the two specimens, because the patient underwent total abdominal hysterectomy with bilateral salpingo-oophorectomy at another center. The treatment of choice of uterine LMS is surgery and the use of adjuvant treatment has limited impact on clinical outcome, and radiotherapy seems only to improve local control, because most recurrences develop at distant sites [6, 10]. The most common chemotherapic agent used for LMSs is doxorubicin, usually associated with ifofosfamide [10, 11]. In this case no adjuvant radiotherapy was administered after total abdominal hysterectomy with bilateral salpingo-oophorectomy, and chemotherapy was started after the detection of lung metastases. In case of metastatic disease, factors that would contribute to the decision of operability would include extent of tumor (TNM), number and type of metastases, disease free interval from the initial diagnosis to the time of metastases, and expected life span [7]. In our case, the decision to proceed with surgical removal of the upper gingiva mass, even in presence of advanced disease, was taken in order to improve quality of life, being chemotherapy and radiotherapy inadequate to control the oral recurrence. Obviously, reconstruction has been achieved using the “simplest” methods, using temporalis muscle flaps, even if a free flap reconstruction is the treatment of choice in order to reconstruct both bone and soft tissue [12, 13]. Bone grafts were not use in order to avoid complication in case of irradiation [14, 15]. Adjuvant radiotherapy was not considered because of resection in free margins with no suspect of residual disease. Nevertheless, local recurrence was observed 5 months after surgical removal so that patient was referred to palliative radiotherapy. In conclusion, even if head and neck metastases from uterine leiomyosarcoma are rare, they have to be suspected in case of rapidly growing haemorrhagic mass. Due to the aggressive growth pattern of the tumor, wide surgical excision in free margins of metastatic lesions could not be sufficient, so that in selected cases, in order to improve local control, the use of adjuvant radiotherapy can be considered. Obviously, this can lead to improving quality of life not influencing clinical outcome.
